# Ketogenic Diet and Microbiota: Friends or Enemies?

**DOI:** 10.3390/genes10070534

**Published:** 2019-07-15

**Authors:** Antonio Paoli, Laura Mancin, Antonino Bianco, Ewan Thomas, João Felipe Mota, Fabio Piccini

**Affiliations:** 1 Department of Biomedical Sciences, University of Padova, 35131 Padova, Italy; 2 Research Center for High Performance Sport, UCAM, Catholic University of Murcia, 30107 Murcia, Spain; 3 Italian Microbiome Project, 35100 Padova, Italy; 4 Sport and Exercise Sciences Research Unit, University of Palermo, 90133 Palermo, Italy; 5 Clinical and Sports Nutrition Research Laboratory (LABINCE), Federal University of Goiás, 74690-900 Goiânia, Goiás, Brazil

**Keywords:** gut microbiota, gut microbiome, intestinal microbiome, ketogenic diet, ketogenic diet and fat

## Abstract

Over the last years, a growing body of evidence suggests that gut microbial communities play a fundamental role in many aspects of human health and diseases. The gut microbiota is a very dynamic entity influenced by environment and nutritional behaviors. Considering the influence of such a microbial community on human health and its multiple mechanisms of action as the production of bioactive compounds, pathogens protection, energy homeostasis, nutrients metabolism and regulation of immunity, establishing the influences of different nutritional approach is of pivotal importance. The very low carbohydrate ketogenic diet is a very popular dietary approach used for different aims: from weight loss to neurological diseases. The aim of this review is to dissect the complex interactions between ketogenic diet and gut microbiota and how this large network may influence human health.

## 1. Introduction

### 1.1. The Human Gut Microbiota and the Microbiome

The human gut microbiota, that means the types of organisms that are present in an environmental habitat, consisting of trillions of microbial cells and thousands of bacterial species [1]. It encompasses ~10^−13^ microorganisms belonging to the three domains of life Bacteria, Archaea and Eukarya and it is involved in several and different functions [2,3]. Microbiome is the collection of the genes and their functions and, due to the new genetic and bioinformatics technologies, the study of the gut microbiome has been radically transformed. The use of the newest platform next generation sequencing (NGS) enables the sequencing of a thousand to million DNA molecules of bacteria in one sequence run (metagenomics) [4] and through this microbial sequencing has been finally possible the understanding of how different microorganisms are present in different tracts of human body [5]. These new omics-technologies allow scientists to discover the role of bacterial genes in human health [6].

Several studies suggest that a mammalian host establishes their core microbiota at birth [7]; the colonization of the gastrointestinal tract by microorganisms, begins within a few hours of birth and concludes around three to four years of age. The nature of the colonic microbiota is driven by several factors such as breast feeding, geographical location, genetics, age and gender [8].

The impact of food (macronutrients) on gut microbiota composition is growing up in interest, especially with respect of specifically dietary fibers. It has been shown that dietary patterns composed by non-refined foods and a high intake of “microbiota accessible carbohydrate” (MACs), are capable to support the growth of specialist microbes producing short chain fatty acids (SCFAs): the prominent energy source for human colonocytes and the signaling key molecules between the gut microbiota and the host [9]. Controversially, the typical pattern of Western diet, high fat-high sugar and low fibers, reduces the production of SCFAs shifting the gastrointestinal microbiota metabolism to the production of detrimental metabolites, favoring the expansion of bacteria associated with chronic inflammation [10].

The composition of the microbiome is influenced by many factors [11] and the stability of the microbiome, reached between two to five years of age, is overlooked by Bacteroidetes, the largest phylum of gram-negative bacteria associated with both beneficial and detrimental effects on health [12,13]. However, the Firmicutes to Bacteroidetes ratio is regarded to be significant for the gut health, the ratio is clearly linked with increasing body mass index (BMI) [14] and the levels of these two dominant bacterial species are known to shift dramatically with aging, especially *Bifidobacterium* and *Lactobacillus* genera [15].

### 1.2. Bioactive Products

The microorganisms living in our gut influence the host through the production of bioactive metabolites, which are able to regulate many biological pathways involved in immunity and energy production. The bacterial population of the large intestine digests carbohydrates, proteins and lipids left undigested by the small intestine. Indigested substances, named “microbiota accessible carbohydrates” (MACs), are represented by the walls of plant cell, cellulose, hemicelluloses and pectin and resistant starch; these polymers undergo microbial degradation and subsequent fermentation [3]. It is really fascinating that the genome of gut bacteria, different from the human genome, encoded several highly specified enzymes able to digest and ferment complex biomacromolecules by hydrolyzing the glycosidic bonds [16,17].

More important, microorganisms have the ability to produce a great amount of B_12_ and K vitamins, essential for human health, especially for the daily vitamin K intake that is most frequently insufficient [18,19].

The prominent end-products of fermentation in the colon are short chain fatty acids (SCFAs) such as butyrate (C_4_H_7_O_2_-) produced especially by Firmicutes, propionate (C_3_H_5_0_2_-) by Bacteroidetes and acetate (C_2_H_4_0_2_) by anaerobes; they represent the greatest source of energy for intestinal absorptive cells. [20,21].

SCFAs contribute to the regulation of the systemic immune function, to the direct appropriate immune response to pathogen and they influence the resolution of inflammation [22].

Moreover, specific bacteria have their own ability to produce many neuroendocrine hormones and neuroactive compounds involved in key aspect of neurotransmission, thus, microbial endocrinology interconnects the science of microbiology with neurobiology. As a matter of fact, γ amino butyric acid (GABA), the major inhibitory neurotransmitter of mammalian central nervous system [23], has been demonstrated to be produced by strains of *Lactobacilli* and *Bifidobacteria*, more specifically by *Lactobacillus brevis*, *Bifidobacterium dentium*, *Bifidobacterium adolescentis* and *Bifidobacterium infantis* [24,25]. *Lactobacillus rhamnosus* has been demonstrated for its therapeutical potential in modulating the expression of central GABA receptors, mediating depression and anxiety-like behaviors [26].

Furthermore, another important mediator of the gut-brain axis is serotonin (5-hydroxytryptamine 5-HT) that is produced by the enterochromaffin cells of the gastrointestinal tract. It is a metabolite of the amino acid tryptophan and plays a pivotal role in the regulation of several functions such as the mood.

The 95% of serotonin is stored in enterochromaffin cells and enteric neurons, while only the 5% is found in the central nervous system. Kim and colleagues found that germ-free mice have a two-fold decrease of the serotonin blood’s level as compared with commonly mice [27].

However, the gut peripheral serotonin is unable to overstep the blood brain barrier; this serotonin acts on lumen, mucosa, circulating platelets and it is grandly implicated in the gut peristalsis and intestinal anti-inflammation [28,29]. Jun Namking and colleagues suggested that the regulation of the peripheral serotonin might be an adequate tool for the treatment of obesity by the increasing of insulin sensitivity [30].

### 1.3. Interindividual Variability of Microbiota

The variability among people and the adaptability of gut microbiota to substantial changes have permitted the manipulation of various external factors, restoring both the biological functions and richness of microbiota [31]. The fact that human microbial community is strictly influenced by diet, and, a good ecological community is connected with a better health, offers a range of opportunity for improving human’s health by changing the microbiota composition through different patterns of diet [32,33,34].

The availability of a huge variety and combination of nutrients promotes the selective enrichment of microorganisms, but both the quality and quantity of the macronutrients have an effect on the structure and function of the microbiome [35].

It has been demonstrated the high fat–high sugar Western diet negatively impacts gut health [36] and a high fat diet is closely related to inflammation [37], however, several studies [38,39,40] suggested the necessity to consider the structure and the function of different fatty acids. De Wit and collaborators [41] showed that specific type of fatty acids affect the gut microbiota in different way and, more recently, it has been said that monounsaturated fatty acid’s (MUFA’s) and polyunsaturated fatty acid’s (PUFA’s) omega 3 may be the control key of low-grade systemic inflammation, gut inflammation and as well as obesity [39].

For these reasons, specialized and restricted dietary regimens adopted as a treatment of some diseases, such as low FODMAP for the irritable bowel syndrome and ketogenic diet for refractory epilepsy, should be investigated for their influence on human microbiota [40,42]. These patterns, by reducing or excluding certain type of foods, may affect positively or negatively the microbiota composition and its related influence on host physiology [43,44,45]. That is the case of very low carbohydrate ketogenic diet (VLCKD), a nutritional approach growing up in interest not only for neurological disorders but also for being a “lose-it-quick-plan” [45,46]. VLCKD, by the drastic reduction of the carbohydrate intake, showed an impairment both on the diversity and richness of gut microbiota [47].

### 1.4. Very Low Carbohydrate Ketogenic Diet (VLCKD)

The very low carbohydrate ketogenic diet (VLCKD) is a dietary protocol that has been used since the 1920 as a treatment for refractory epilepsy [48] and it is currently getting popularity as a potential therapy for obesity and related metabolic disorders [49]. Due to the typical pattern of VLCKD, a hot topic in research and an evolving area of study has been the effect of ketogenic diet on the gut microbiome [50,51,52,53].

Ketogenic diet permits a very low carbohydrate consumption (around 5% to 10% of total caloric intake or below 50 g per day), as a mean to enhance ketone production [54].

Originally, VLCKD had been used as a treatment for epileptic patients that failed to respond to anticonvulsant medication [55]. Currently it has become popular for its benefits extended to neurodegenerative diseases, metabolic diseases and obesity [45]. Recently, VLCKD has been demonstrated to be a powerful tool for some neurodegenerative disease such as autism spectrum disorder (ASD), Alzheimer’s disease [46], glucose transporter 1 deficiency syndrome [56] and auto immune multiple sclerosis (AIMS) [57]. Given the fact that VLCKD is a highly restricted dietary pattern, nowadays, there has been the necessity of formulating new features of the VLCKD, such as the popular modified Atkins diet (MAD) and low glycemic index diet (LGIT) [58,59].

These new patterns have been demonstrated as a successful tool able to reduce seizure symptoms, as well as they reveal a similar outcome, with lower side effects, while compared to the traditional regimes of VLCKD [60,61,62]. LGIT, different from the modified Atkins regime, involves avoiding high glycemic carbohydrates to stabilize blood glucose since it has been shown that stable glucose levels are associated with seizure control [63]. Due to the MAD and LGIT people may choose in a more flexible way the meal they want to consume, they do not have to calculate the specific ketogenic ratio but they may focus on ensuring sufficient and appropriate fats, both in quantity and quality.

### 1.5. Physiology of Ketosis

The very low carbohydrate ketogenic diet (VLCKD) share several pathways that have been found during fasting state [64]. After several days of drastically reduction of carbohydrate intake, at least <20 g/d or 5% of total daily energy intake, the glucose in the body results insufficient for both fat oxidation (oxaloacetate in tricarboxylic acid cycle TCA) and energy required for the central nervous system forcing the organism to use fats as a primary fuel source [65].

However, fat free acids do not provide energy for the brain because they are not capable to overstep the blood brain barrier: This energy is provided by ketone bodies.

Ketone bodies, 3 hydroxybutyrate (3HB), acetate and acetoacetate (AcAc) are produced in the liver through the process of ketogenesis. Ketogenesis takes place especially in the mitochondria of liver cells where fatty acids reach the mitochondria via carnitine palmitoyltransferase and then breaks down into their metabolites, generating acetyl CoA. The enzyme thiolase (or acetyl coenzyme A acetyltransferase) converts two molecules of acetyl-CoA into acetoacetyl-CoA. Acetoacetyl-CoA is then converted to HMG-CoA due to the enzyme HMG-CoA synthase. Lastly, HMG-CoA lyase converts HMG-CoA to acetoacetate, which can be decarboxylated in acetone or, via β-hydroxybutyrate dehydrogenase, transformed in β-hydroxybutyrate.

The less abundant ketone body is acetone while 3HB plays a main role in the human body under low carbon hydrate diet [66].

**Figure 1:**
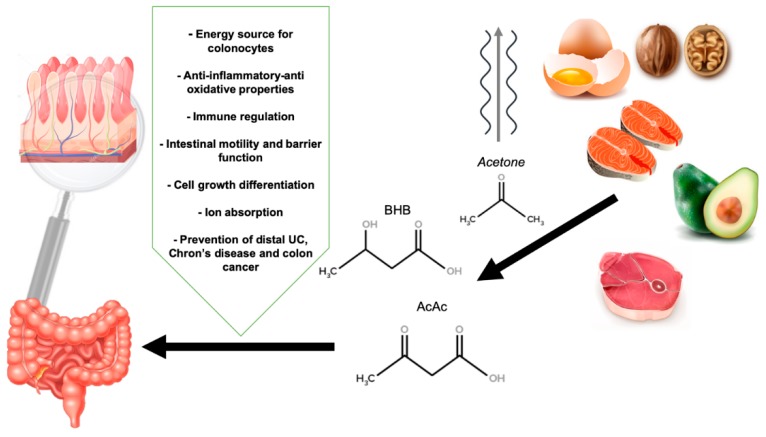
The influence of a very low carbohydrate ketogenic diet and ketone bodies in gut health. BHB: β-hydroxybutyrate, AcAc: Acetoacetate.

The global view of how VLCKD may influence the gut’s health is shown in Figure 1.

## 2. Methods

We performed a systematic review from February to March 2019; we used the electronic databases Pubmed, (MEDLINE) and Google scholar. We adopted the MeSH term through the function “MeSH Database” within Pubmed. The terms combined with Boolean operators AND, OR, NOT have been “gut microbiota”, “gut microbiome”, “intestinal microbiome”, “ketogenic diet”, with “ketogenic”, “fat”. Eligibility criteria included full-text articles, written in English, available online from 2015 to 2019; specific studies in which authors investigated the effect of the ketogenic diet on gut microbiota and declared no conflict of interest. We decided to include both in vivo and in vitro studies, ranging from randomized controlled trials to case-control and, to emphasize the effects of diet in “fixed” conditions, we included as well animal studies.

## 3. Results

### How VLCKD Affects the Gut Microbiome

As the ketogenic diet seems to gain consensus [63], little is still known about its impact on the gut microbiota.

Only few experimental studies sought the relationship between VLCKD and gut microbiome [47,50,52,53,67,68,69,70] investigating how VLCKD impacts composition and characteristics of intestinal microorganisms. The effects of VLCKD on gut microbiome have been explored in mice and humans with mixed results. Our systematic review included nine studies and the major findings have been schematically reported (Table 1).

Recently, [53] it has been explored the role of VLCKD on gut microbiota related to the anti-seizure effect on mice. In this study, they found that mice, within four days of being on a diet, had significant changes in gut bacterial taxonomy. Two species of bacteria, *Akkermansia* and *Parabacteriodes* were significantly increased in mice that were fed ketogenic diets and gnotobiotic colonization with these microorganisms revealed an anti-seizure effect in germ-free mice or treated with antibiotics.

The increase of these two bacteria species in the gut led to a decreased production of γ-glutamyl transpeptidase by the gut microbiome, the enzyme catalyzes the transfer of γ-glutamyl functional groups from molecules such as glutathione to an acceptor that may be an amino acid forming glutamate [71].

Moreover, they observed a decrease in subset of ketogenic γ-glutatamylated (GG) amino acids (i.e., γ-glutamyl-leucine) both in the gut and blood. GG amino acids are supposed to have transport properties across the blood–brain barrier, different from non-γ-glutamylated forms [72]: This property is involved in glutamate and GABA biosynthesis [73].

This fact, in turn, had the effect of increasing the ratio of GABA to glutamate in the brain of mice. The researchers suggested that VLCKD-microbiota-related limitation in GG amino acids plays a pivotal role on anti-seizure effect, confirmed by the previous studies showing GGT activity to modify the electrical activity of seizure [53].

The ketogenic diet, composed by short fatty acids SFAs, monounsaturated fatty acids MUFAs and polyunsaturated fatty acids PUFAs, has been provided for 16 weeks by Ma and colleagues [51] and it reveals that mice had a variety of neurovascular improvement strictly linked to a lower risk of developing Alzheimer’s disease. These beneficial effects may be connected with the changing on gut microbiota composition, more specifically with the growing quota of beneficial bacteria, Akkermansia Muciniphila and Lactobacillus, which have the ability of generating short chain fatty acids SCFAs. Interestingly, they found a reduction in pro-inflammatory microbes such as Desulfovibrio and Turicibacter. The VLCKD however, decreased the overall microbial α diversity due to the low carbohydrate (complex carbohydrate) content of diet, which is fundamental for the microorganism in order to breakdown them and producing energy [52].

A 2016 study [67] investigated whether or not a VLCKD could ensure benefits in the gut microbiome in murine model of autism. The authors administrated a VLCKD for several days (10–14) observing changes in gut microbiome; they concluded that the VLCKD had an “anti-microbial” effect by decreasing the overall richness of microorganisms both in cecal and fecal matter, and as well as improved the ratio of Firmicutes to Bacteroides species. A lowered firmicutes: bacteroides ratio is common in ASD and the VLCKD, by improving this ratio, was able to enhance ASD behavioral symptoms. Lastly, different from the above-mentioned studies, the VLCKD decreased the number of *A*. *muciniphila* bacteria species, resulting in similar levels to those found in the control groups.

It has been described the connection between microbiome, VLCKD and the potential role playing in multiple sclerosis (MS) [52]. A common attribute of the AIMS is the damage and affliction of “colonic bio-fermentative function”. The fermentative process which allow the production of beneficial byproducts such as SFCA, is impaired, thus, the dysbiotic colonic bacteria ferment foods into dangerous compounds affecting the organism. The VLCKD completely restored the microbial biofermentative mass and normalizing the concentration of the colonic microbiome. The authors [52] showed a biphasic effect of VLCKD: first there has been a dramatic decrease in richness and bacterial diversity, but, after 12 weeks, bacterial concentration began to recover back to baseline and, after 23–24 weeks, it showed a significant increase in bacterial concentration above baseline.

A study in children by Xie and colleagues [68], investigated the connection between microbiome and refractory epilepsy in 14 epileptic and 30 healthy infants. Patients with epilepsy demonstrated an imbalance of gut microbiota before starting the VLCKD. The authors found a higher amount of pathogenic proteobacteria (Escherichia, Salmonella and Vibrio), which significantly decreased after VLCKD treatment and an increase of Bacterioidetes both in healthy subjects and in patients. Bacteroides spp. are strictly connected with the digestion and metabolism of high-fat nutrients and the regulation of the secretion of 6–17 interleukins in dendritic cells, which is connected with the seizure effects on epileptic patients [74]. Researchers suggest that VLCKD can reduce these symptoms by developing changes on microbiota diversity.

Zhang et al. sought the differences in the microbiota of pediatric patients fed a ketogenic diet [69]. They investigated the difference between responders (seizure frequency was reduced or stopped) and non-responders (no effect on seizure). They found increased amount of *Bacteroides* and decreased amounts in *Firmicutes* and *Actinobacteria*, in responders. On the other hand, *Clostridia, Ruminococcus* and *Lachnospiraceae* (Firmicutes phylum) increased in non-responders. These data demonstrated that ketogenic diet alters the gut microbiome of pediatric patients, suggesting that the gut microbiome should be taken into account as a biomarker of efficacy of anti-seizure treatment. As regard patients affected by Glucose Transporter 1 Deficiency Syndrome [50], it has been showed a significant increase in *Desulfovibrio* spp. in six patients, after 3 months of intervention. *Desulfovibrio* spp is a genus belonging to a heterogeneous group of sulfate-reducing, motile, anaerobic bacteria related to the inflammatory status of the gut layer mucosa [75]. Authors suggested that in case of dysbiosis, it might be a good option the use of an extra-supplementation with pre or probiotics to maintain the “*ecological balance*” of gut microbiota [50].

Recently, a study in epileptic children found a reduction of *Bifidobacteria*, as well as *E. rectale* and *Dialister*, which are correlated with health promoting benefits such as the prevention of colorectal cancer, IBS and necrotizing entercolitis [76]. Researcher identified a relative abundance of *Actinobacteria* and *Escherichia coli* that may be due to the VLCKD restricted on carbohydrate. It should be stressed that through the analysis of the 29SEED subsystem, scientists revealed a depletion of those pathways responsible of the degradation of carbohydrates [70].

## 4. Discussion

### 4.1. Friend or Enemies?

**Figure 2:**
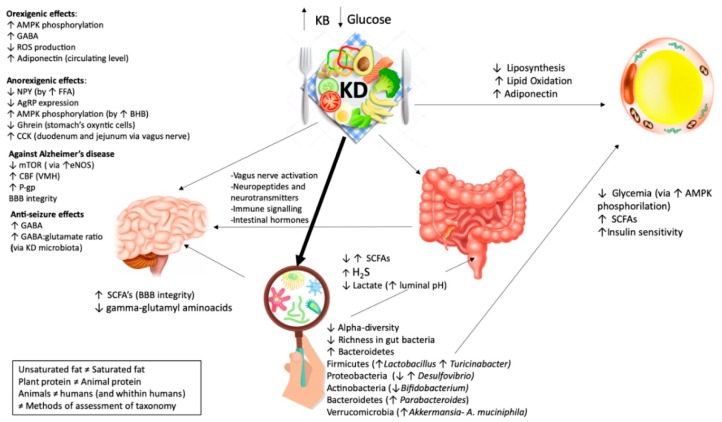
Effects of ketogenic diet on different tissues and the microbiome. KD has a contradictory role on hunger but the net effect is anorexigenic. KD Exerts orexigenic effects: The increase of brain GABA (γ-aminobutyric acid) through BHB (β-hydroxybutyric acid); the increase of AMP (adenosine monophosphate -activated protein) phosphorylation via BHB; the increase of circulating level of adiponectin; the decreases of ROS (reactive oxygen species). KD Exerts anorexigenic effect: the increase of circulating post meal FFA (free fatty acids); a maintained meal’s response of CCK (cholecystokinin); a decrease of circulating ghrelin; a decrease of AMP phosphorylation; a decrease of AgRP (agouti-related protein) expression. KD has positive effects on Alzheimer’s disease through: an increase levels of CBF (cerebral blood flow) in VMH (ventromedial hypothalamus); a decrease expression of mTOR (mammalian target of rapamycin) by the increase of the level of eNOS (endothelial nitric oxide synthase) protein expression; an increased expression of P-gp (P-glycoprotein), which transport Aβ (amyloid-β) plaques; an improvement of BBB’s (blood–brain-barrier) integrity. KD has beneficial effects on epileptic seizure by the modulation of hippocampal GABA/glutamate ratio. It exerts anti-seizure effects through: An increase level of GABA, an increase content of GABA: glutamate ratio. KD plays a main role on fat loss. It exerts positive effects on adipose tissue through: a decrease of liposynthesis, an increase of lipid oxidation and an increase in adiponectin. KD has a contradictory role on microbiome. KD generally exerts its effect through: a decrease in α diversity (the diversity in a single ecosystem/sample) and a decrease in richness (number of different species in a habitat/sample). KD influences the gut health through metabolites produced by different microbes: an increase/decrease in SCFA (short chain fatty acids), an increase in H2S (hydrogen sulfide) and a decrease in lactate. KD to microbiome to the brain: KD may influence the CNS (central nervous system) not only directly but also indirectly. The KD effects on the brain are supposed to be mediated by microbiota through an increase of SCFAs and a decrease of γ-glutamyl amino acid. *A. muciniphila* and *Lactobacillus* are known as SCFAs producers. SCFAs are transported by monocarboxylase transporters expressed at BBB. *Desulfovibrio* has the ability to produce hydrogen sulfide and, as a consequence, impair intestinal mucosal barrier. A reduction in *Desulfovibrio* and an enhancement in *A. muciniphila* and *Lactobacillus* may facilitate BBB and neurovascular amelioration. KD to microbiome to the adipose tissue: KD may indirectly influence the adipose tissue by the microbiota through a decrease in glycemia via adenosine monophosphate-activated protein kinase (AMPK) phosphorylation, an increase in insulin sensitivity and an increase in SCFAs. The great amount of *A. muciniphila* and *Lactobacillus* spp. led to the reduction of body weight and glycemia. It has been demonstrated that patient with type 2 diabetes, treated with metformin, revealed higher level of *A. muciniphila*, may be to the ability of metformin on decreasing body weight by the activation of AMPK pathways (amp-activated protein kinase). *A. muciniphila* is related with the enhancement of insulin sensitivity and *Lactobacillus* may be playing the same effects through SFCAs production: Several studies showed that *Lactobacillus* is strictly connected with body weight loss.

All the papers that have been chosen for depicting the crossing mechanisms, revealed supposed connections between gut microbiome, ketogenic diets and systemic effects. Some findings are demonstrated through “omics” analyses, some are only assumed. As it can be seen, there are several and controversy findings suggesting the necessity of a deeper understanding. The picture (Figure 2) aims to highlight the supposed major effects of ketogenic diet on different tissues and gut microbiota, along with how tissues may be influenced by gut microbiota diversity.

### 4.2. Factors Affecting Microbiota during a VLCKD: What Should We Consider?

#### 4.2.1. Fats

The optimal composition of a VLCKD considers both high saturated and mono-polyunsaturated fats [54], whilst the Western diet is rich in saturated-trans fats and poor in mono-polyunsaturated fats [77].

A recent systematic review concluded that diets high in saturated fatty acids led to negative effects on the gut microbiota [78]. The authors observed that diets rich in highly monounsaturated fats affected negatively the gut microbiota decreasing bacteria richness, while diets rich in polyunsaturated fatty acids (with opposite effects when comparing omega 3 vs. omega 6 fats) did not change richness and diversity. However, to notice that only a few studies have been conducted with NGS methods or shotgun sequencing, these new technologies deliver accurate data by avoiding experimental pitfalls and biases created by the “old fashioned” sequencing methods [79]. Recently, a randomized controlled trial study [80] has revealed that a diet with a high content in fat increased *Bacteroides* while reducing the number of butyrate producers (*Faecalibacterium* and *Blautia* bacteria) compared with a middle-lower-fat group. The differences in fecal SCFA could be explained by the high content of carbohydrates in the middle to low-fat diets, made up of resistant starches that have been broken down and fermented. It has to be stressed that the source of fat came from soybean oil, which is highly rich in omega 6 polyunsaturated fatty acids [81]; a higher omega-6: omega-3 long-chain PUFA ratio is associated with many health risks and chronic state of inflammation [82,83,84]. Another RCT study [85] showed that a supplementation with omega 3 PUFA did not disclose any taxonomic changes both in α and β diversity (at family and genus levels) including short-chain fatty acid producers.

According to these results, different studies demonstrated that each type of fatty acid may induce different effects: The saturated fats (palm oil) induce higher liver triglyceride content in mice, as opposed to polyunsaturated fats (olive oil) [41]. Moreover, genetically modified mice, characterized by the ability of producing omega 3 (PUFAs) and fed with high fat and high sugar diet, showed a higher microbial diversity and a normal gut layer function in the distal intestine, different from non-modified-mice fed with the same macronutrients [86].

The source of fats (omega 6: Omega3, PUFAa and MUFAs) and their own quality should be highly considered when performing a very low carbohydrate dietary plan and as well as when giving general nutritional advices.

#### 4.2.2. Sweeteners

An area of controversy in the ketogenic diet is the consumption of artificial sweeteners replacing natural sugars. Several evidences demonstrated that artificial sweeteners have a negative impact on both host and gut health. Nettleton at al. found that low calorie sweeteners, such as acesulfame potassium (Ace-K) and sucralose, disrupted the structure and function of gut microbiota and gut mucosa [87]. More recently Qiao-Ping Wang investigated, through the use of NGS, the effects of non-nutritive sweeteners (NNSs) on the gut microbioma of mice at the organism level; the study reveals that artificial sweeteners has bacteriostatic effects and as well as change the composition of microbiota [88]. These findings, according to the fact that the routine consumption of NNSs may increase the risk of cardiometabolic diseases [89], suggested that these chemical substitutes may be detrimental for human health and should be avoided [90]. However, recently, the use of stevia (also called *Stevia rebaudiana)* has been widely adopted as a non-nutrient but natural sweeteners. The use of Stevia lowered insulin and glucose level in 19 healthy lean and 12 obese individuals and left them satisfied and full after eating, despite the lower calorie intake [91]. Accordingly, Sharma and colleagues [92] showed a reduction of cholesterol level, triglyceride, low-density lipoprotein (LDL) and an enhancement of high-density lipoprotein (HDL) on 20 hypercholesterolemic women consuming stevia extracts. In a 2008 review, authors suggest that there are not enough information concerning the effect of stevia on gut microbioma [93], whilst others reported a possible link between nonnutritive sweeteners, including stevia, and the disruption of beneficial intestinal flora [94].

Given the fact that there is no explicit data available on gut microbiome, but, The Food and Drug Administration (FDA) considered it as “generally safe” [95], stevia might slightly be used in place of artificial and chemical sweeteners, within coffee, tea or in a unsweetened yogurt. However, further investigation need to be done considering the effect of low calorie sweeteners on gut and human health.

#### 4.2.3. Pre and Probiotics

A proper suggestion for maintaining a healthy gut microbiota during the ketogenic diet may be the use of pre and probiotics: Increasing evidences [96,97] demonstrate their positive benefits. The major source of prebiotics is represented by fructo-oligosaccharides, inulin, lactulose galacto-oligosaccharides and trans-galacto-oligosaccharides [98]. Fermentation of prebiotics by gut microbiota produces SCFAs, which positively modulate the composition of microbiota (by increasing intestinal bifidobacteria and lactic acid bacteria), providing an energy source for colonocytes [99]. Differently, probiotics are living bacteria (especially from the genera *Bifidobacterium* and *Lactobacillus)* and yeasts that, when administrated in an adequate amount, show positive effect on human health; they are usually added to yogurts or found in “specialty food” [100,101,102]. It has been reported [103,104] that foods enriched with these microorganisms are able to recovery and improve gut microbiota, reaching the state of eubiosis. Cultured-milk products (kefir, Greek yogurt), traditional buttermilk, water kefir, fermented cheese, sauerkraut, kimchi, miso, kombucha and pickles contain several and different “friendly bacteria” such as *Lactobacillus acidophilus*, *Lactobacillus delbrueckii* subsp. *bulgarius*, *Lactobacillus reuteri*, *Saccharomyces boulardii* and *Bifidobacterium bifidum* [105,106,107,108].

However, despite the growing interest on fermented foods, there is a lack of epidemiological studies [104] and the majority have focused only on yogurt and cultured dairy foods [109,110]. The paucity arises from the difficulty of understanding if health benefits come from the fermentation operated by microbes or other bioactive compounds. As regard the usefulness of fermented foods during a VLCKD, they represent an excellent and palatable source of dietary fiber and essential micronutrients [111], which should be moderately provided during a VLCKD.

In our opinion since foods that have undergone deep fermentation seem to improve the gut microbiome diversity and gut health index [112] adding small portions of fermented foods to the diet may be a useful prebiotic/probiotic supplementation as well as an effective aid to digestion. A caveat should be done: It is mandatory to verify that fermented foods and beverages are able to not modify in a significant manner glycaemia and insulinaemia while maintaining a sufficient ketonemia.

It has been recently shown that parmesan (an Italian hard and dry cheese), contains “friendly bacteria” acting as probiotics and able to colonize and live in the gut of those individuals who daily consume it [113]. Thus, the moderate consumption of a high-fat fermented food is well recommended for human gut and human health.

#### 4.2.4. Proteins

Several considerations have to be done to the different impact of different protein on gut microbiome.

The source and type of protein must be considered, especially in the field of sports, in which the intake of protein within VLCKD is fundamental to maintain lean body mass [114].

Several studies investigated how and how much different kind of protein (plant versus animal) modify microbiome [115,116,117], showing that, even though high protein diet generally impair gut health (decrease abundance and change composition) [118], several and disparate effects appear on the gut microbiota [119].

Plant-derived protein, such as mung bean protein (as a part of high fat diet), increased Bacteroidetes while decreasing *Firmicutes* as well as pea protein increased strains of Bifidobacterium and lactobacillus [115].

These studies demonstrated that plant-derived protein get better benefits on gut microbiome along with positive effects on the host metabolism.

To note that we did consider that no studies investigated how protein have been processed, such as thermal treatment, and the effect of the processing treatment on microbiome composition.

During a period of VLCKD, we recommend the use of a source of plant protein (veg protein) since these are more beneficial in terms of health gut microbiota.

## 5. Conclusions, Perspective and Future Research

In the recent years, the interest regarding the benefits of ketogenic diets is growing up and expanding well beyond the seizure control. Ketogenic diet, as well as the more flexible and less restrictive regimens MAD, LGIT is commonly adopted for weight loss in both obese patients and athlete populations. Bacteria taxa, richness and diversity are strictly influenced by ketogenic diet. A few human and animal studies have shown different results demonstrating positive effects on reshaping bacterial architecture and gut biological functions, while others reporting negative effects as a lowered diversity and an increased amount of pro-inflammatory bacteria.

Nevertheless, short period studies and with specific disease conditions have been carried out [50,52,67,68], limiting generalization to the overall population. Additionally, the microbiota of many environments may be highly variable and its plasticity could be dependent on past and specific dietary patterns [120]. In agreement with these considerations, Healey and colleagues concluded that because of the high variability among people of microbiome composition, it is actually difficult to identify how microbiota may change the diversity in relation to a specific dietary pattern [121]. According to different authors [50,70], there is the necessity to find better strategies to maximize the benefit of VLCKD. It may be useful implementing VLCKD with specific pre and probiotics, which has been found to be drastically reduced during VLCKD [50]. Additionally, promising evidence comes from randomized control trials suggesting that quality dietary fats highly affects the gut microbiota composition. Diets with a high fat content and good quality of polyunsaturated fats and plant-derived protein are able to maintain normal gut function [80,86]. In parallel, the abolition of artificial sweeteners [90] should be recommended to avoid negative effects on general health caused by alteration of gut microbiota. It has been suggested that a supplementation with prebiotics, such as inulin, lactulose, fruttooligosaccharides (FOS) and galactooligosaccharides (GOS) that increases Bifidobacteria, may prevent undesired changes in the gut microbiota [122].

Nonetheless, it is essential to point out that the modified microbiota composition, changed by VLCKD, plays a pivotal role on the itself activity of VLCKD [53,67,68]; the changes have been demonstrated to be necessary in order to provide positive effects such as the anti-seizure effect and amelioration of neurovascular function [53,69,70].

Although there are still many questions limiting the practical research on microbiome, several new developments carried on advancement in this field. Integration of omics science with the newest metagenomic methods of microbiota assessment (next generation sequencing, shotgun sequencing 16S rRNA) shall be helpful to define healthy versus unhealthy microbial operational taxonomic units (OTUs). For this purpose, the Italian Microbiome Project (http://progettomicrobiomaitaliano.org) focuses his research on the advantages and disadvantages that may arise from the genes of bacterial origin, by combining bioinformatic tools with algorithms to better link microbiota data to human health outcomes. Recently, it has been developed a machine e-learning algorithm that is able to predict a specific post-prandial glycemic response by analyzing microbiome profiling [123,124].

The observations that a ketogenic diet can modulate and reshape gut microbiota represents a potential and promising future therapeutic approach. VLCKD has been demonstrated to be a powerful tool and needs to be further refined and well formulated considering its impact on gut health. In conclusion, further research with long-term clinical trials has to be performed in order to establish safer and healthier specific dietary interventions for patients.

Take Home Message:

Practical recommendations to preserve gut health during a VLCKD:

- Introduce the use of whey and plant proteins (i.e., pea protein);
- Reduce the intake of animal protein;
- Implement fermented food and beverages (yoghurt, water and milk kefir, kimchi, fermented vegetables);
- Introduce properly prebiotics and specific probiotics (if needed);
- Reduce omega 3 to omega 6 fatty acids ratio (increase omega 3 while decreasing omega 6);
- Introduce an accurate quantity and quality of unsaturated fatty acids;
- Avoid artificial sweeteners (stevia?) and processed foods;
- Test your microbiome if needed (analysis of 16S rRNA to identify biodiversity and richness).

It is mandatory to verify that fermented foods and beverages and proteins should not modify (in a significant manner) glycaemia and insulinaemia while maintaining a sufficient ketonemia.

We need to remember as well as that the modified microbiota composition induced by VLCKD, plays a pivotal role on the itself activity of diet.

## Figures and Tables

**Table 1:** Main findings of the effects of Ketogenic diet (KD) on gut microbiome.

	Subjects	Subjects Characteristics	Duration	Type of KD	Measured KBs (Y/N)	KBs’ Level	Genome Analysis Technique	Main Findings of Bacteria Changes
**Tagliabue et al. (2017)** [50]	6 patients (3 females 3 males) pre-post	Glucose Transporter 1 Deficiency Syndrome	3 months	First 1:1 ratio with gradual increase of 2:1, 3:1 and or 4:1 KD ratio	Ketonuria	Not mentioned	DNA extraction RT-qPCR analysis	INCREASE *Desulfovibrio* spp.
**Swidsinki et al. (2017)** [52]	25 MS patients and 14 controls	Auto Immune Multiple Sclerosis	6 months	>50 g carbohydrate, >160 g fat, <100 g protein	Ketonemia and ketonuria	β-hydroxybutyric acid ≥ 500 μmol/L; acetoacetate ≥ 500 μmol/L	FISH with ribosomial RNA derived probes	DECREASE β-diversity, DECREASE substantial bacteria groups after two weeks, after six months completely recover the concentration to baseline
**Newell et al. (2017)** [67]	25 juvenile male C57BL/6 (B6) and 21 BTBR mice	Autism Spectrum Disorder	10–14 days	75% kcal fat	Ketonemia	β-hydroxybutyric acid 5.1 ± 0.8 mmol/L	DNA extraction RT-qPCR analysis	DECREASE in total bacterial content both in cecal and fecal analysis, DECREASE *A*. *muciniphila* both in cecal and fecal matter, INCREASE Enterobacteriaceae in fecal matter
**Burke et al. (2019)** [47]	10 LCHF, 10 PCHO, 9 HCHO pre-post	Elite race walkers	3 weeks	78% fat, 2.2 g/kg BM/day protein, <50 g carbohydrate	Ketonemia	β-hydroxybutyric acid ≥ 1.0 mmol/L	16S rRNA-gene amplicon sequencing	INCREASE in *Bacteroides* and *Dorea* spp. DECREASE in *Faecalibacterium* spp.
**Lindefeldt et al. (2019)** [70]	12 children (parents as controls) pre-post	Therapy-resistant epilepsy	3 months	4:1 in 7 children, 3.5:1 in 2, and 3:1 in 3 KD ratio	Ketonemia	β-hydroxybutyric acid 0.3 ± 0.2 mmol/L	Shotgun metagenomic DNA sequencing	DECREASE in abundance of bifidobacterium, *E. rectale*, *E. dialister*, INCREASE in *E. coli*, changes in 29 SEED subsystem: reduction of seven pathways of carbohydrate metabolism
**Olson et al. (2018)** [53]	Juvenile SPF wild-type Swiss Webster mice, GF wild type SW mice, SPF C3HeB/FeJ KCNA1 KO mice	6 Hz induced seizure model of refractory epilepsy	3 weeks	6:1 KD ratio	Ketonemia (liver, colon, intestine) and normalized to SPF (specific-pathogen free)	β-hydroxybutyric acid (different levels accepted)	16S rRNA-gene amplicon sequencing	DECREASE in α diversity, INCREASE *A.* *muciniphila*, Parabacteroides, Suttarella and Erysipelotrichaceae
**Zhang et al. (2018)** [69]	20 patients (14 males 6 females) pre-post	Refractory epilepsy	6 months	4:1 KD ratio (plant fat 70%, 1 g/kg BM/day from animal source	Ketonemia	β-hydroxybutyric acid 2.85 ± 0.246 and 3.01 ± 0.238 mmol/L (effective and ineffective group)	16S rRNA-gene amplicon sequencing	DECREASE in α diversity, Firmicutes, Actinobacteria, INCREASE in Bacteroidetes
**Ma et al. (2017)** [51]	C57BL/6 male mice	Healthy mice	4 months	75% fat (saturated, monounsaturated, polyunsaturated), 8.6% protein, 3.2% carbohydrates	Ketonemia	β-hydroxybutyric acid around 1.5 mmol/L	16S rRNA-gene amplicon sequencing	DECREASE in diversity, INCREASE *A. muciniphila*, *Lactobacillus*, DECREASE *Desulfovibrio*, *Turicinabacter*
**Xie et al. (2017)** [68]	14 patients and 30 healthy infants	Refractory epilepsy	1 week	lipid-to-non-lipid ratio of 4:1 (40% medium chain, 60% long chain), 60–80 kcal/kg per day, 1–1.5 g/kg protein	Not mentioned	Not mentioned	16S rRNA-gene amplicon sequencing	DECREASE Proteobacteria (*Cronobacter*), INCREASE Bacteroidetes (*Bacteroides*, *Prevotella*), *Bifidobacterium*

KD: Ketogenic diet; RT-qPCR: Real-time quantitative polymerase chain reaction; MS: Multiple Sclerosis; FISH: Fluorescent in situ hybridization; rRNA: ribosomial ribonucleic acid; SPF: specific-pathogen-free; SW: Swiss Webster.
